# Supplementary material for: AGSAM: Agent-Guided Segment Anything Model for Automatic Segmentation in Few-Shot Scenarios
Source: Bioengineering (Basel). 2024 Apr 30;11(5):447. doi: 10.3390/bioengineering11050447 (PMC11118214; doi:10.3390/bioengineering11050447)
Supplement: Supplementary file 1 [file bioengineering-11-00447-s001.zip › bioengineering-2925598-supplementary.pdf]

# Supplementary Material

## 1. Feature Augmentation Convolution Module (FACM)

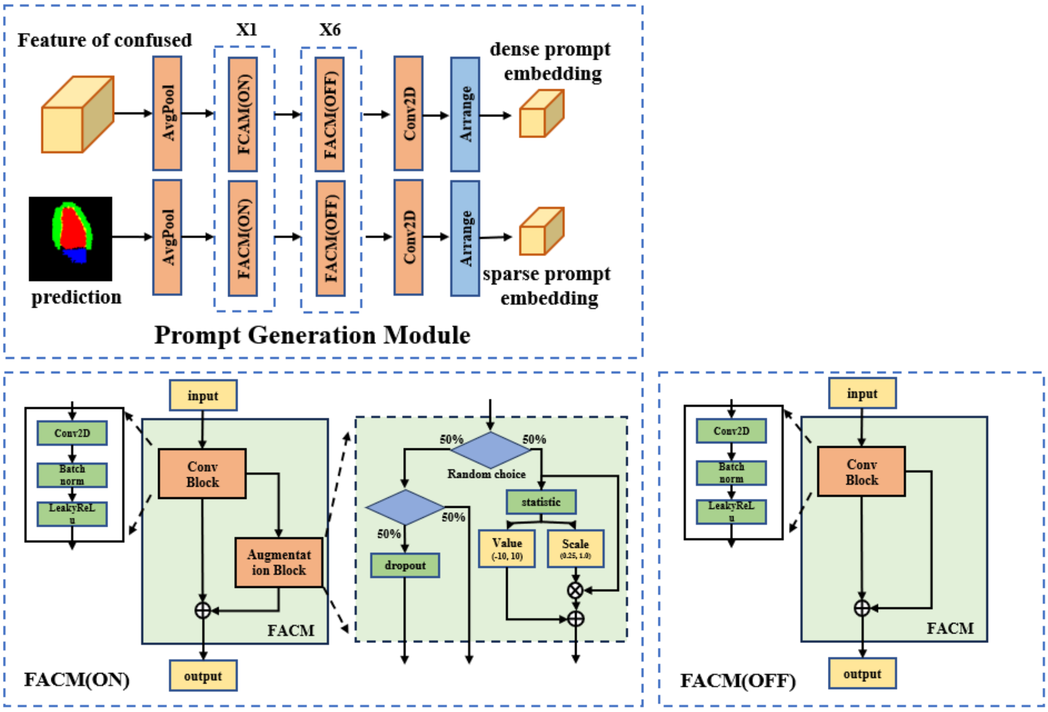


**Figure S1. | Architecture of Prompt Generation Module and Feature Augmentation Convolution Module.** The FACM has two states: one is with the Augmentation Block enabled (ON), and the other is with the Augmentation Block disabled (OFF). When the ON state is active, the structure involves random linear suppression and dropout. When the OFF state is active, the original branch becomes an identity mapping.

| **Prompt Generation Module** | | | | |
|---|---|---|---|---|
| Layer id | Module | Switch | Kernel size | Stride |
| **1** | AvgPool | / | 3 | 2 |
| **2** | FACM | ON | 3 | 2 |
| **3** | FACM | OFF | 3 | 2 |
| **4** | FACM | OFF | 3 | 2 |
| **5** | FACM | OFF | 3 | 1 |
| **6** | FACM | OFF | 3 | 1 |
| **7** | FACM | OFF | 3 | 1 |
| **8** | FACM | OFF | 3 | 1 |
| **9** | Conv2D | / | 1 | 1 |

**Table S1 Architecture of Prompt Generation Module**

## 2. Experiment setup

| Hyper parameters | Comparative study | | | | | | | | |
|---|---|---|---|---|---|---|---|---|---|
| | 1-shot | 2-shot | 4-shot | 6-shot | 8-shot | 12-shot | 16-shot | 20-shot | Full |
| **Image size** | 256*256 | 256*256 | 256*256 | 256*256 | 256*256 | 256*256 | 256*256 | 256*256 | 256*256 |
| **Batch size** | nnSAM:4 MambaUnet:1 Other:8 | nnSAM:4 MambaUnet:1 Other:8 | nnSAM:4 MambaUnet:1 Other:8 | nnSAM:4 MambaUnet:1 Other:8 | nnSAM:4 MambaUnet:1 Other:8 | nnSAM:4 MambaUnet:1 Other:8 | nnSAM:4 MambaUnet:1 Other:8 | nnSAM:4 MambaUnet:1 Other:8 | nnSAM:4 MambaUnet:1 Other:8 |
| **Epoch** | 50 | 50 | 50 | 50 | 50 | 50 | 50 | 50 | 50 |
| **Learning rate** | SegFormer:1e-5 Other:1e-4 | SegFormer:1e-5 Other:1e-4 | SegFormer:1e-5 Other:1e-4 | SegFormer:1e-5 Other:1e-4 | SegFormer:1e-5 Other:1e-4 | SegFormer:1e-5 Other:1e-4 | SegFormer:1e-5 Other:1e-4 | SegFormer:1e-5 Other:1e-4 | SegFormer:1e-5 Other:1e-4 |
| **Data augmentation** | Random rotate ±10° | Random rotate ±10° | Random rotate ±10° | Random rotate ±10° | Random rotate ±10° | Random rotate ±10° | Random rotate ±10° | Random rotate ±10° | Random rotate ±10° |
| **Fusion rate SAM：Agent** | 0.9 | 0.9 | 0.9 | 0.75 | 0.75 | 0.75 | 0.7 | 0.7 | 0.5 |

**Table S2. Hyper parameters of different experiments.** Image size: the resolution of images after uniform processing, Batch size: the amount of data in each batch during training, Epoch: the total number of training epochs, Learning rate: the initial learning rate at the beginning of training, Data augmentation: the content of data augmentation, Fusion rate: the optimal ratio between agent and SAM for AGSAM's best results.

| Hyper parameters | Ablation study | | |
|---|---|---|---|
| | FCN | DeepLabv3 | Unet++ |
| **Image size** | 256*256 | 256*256 | 256*256 |
| **Batch size** | 8 | 8 | 8 |
| **Epoch** | 50 | 50 | 50 |
| **Learning rate** | 1e-4 | 1e-4 | 1e-4 |
| **Data augmentation** | Random rotate ±10° | Random rotate ±10° | Random rotate ±10° |
| **Fusion rate SAM：Agent** | 0.9 | 0.9 | 0.9 |

**Table S3. Hyper parameters of different experiments.**

# 3. Comparison of performance among different methods in CAMUS

| Method | Metrics | | | | | | | | | | | | | | | |
|---|---|---|---|---|---|---|---|---|---|---|---|---|---|---|---|---|
| | SEN | | | | | | | | SPEC | | | | | | | |
| | training sample size(n) | | | | | | | | | | | | | | | |
| | 1 | 2 | 4 | 6 | 8 | 12 | 16 | 20 | 1 | 2 | 4 | 6 | 8 | 12 | 16 | 20 |
| **FCN** | 0.6290 | 0.7626 | 0.6935 | 0.7537 | 0.7523 | 0.7985 | 0.8070 | 0.7975 | 0.9425 | 0.9548 | 0.9636 | **0.9862** | 0.9882 | 0.9882 | 0.9890 | **0.9897** |
| **deeplabv3** | 0.5354 | 0.7283 | 0.7034 | 0.7327 | 0.7383 | 0.7962 | 0.7984 | 0.8132 | 0.9751 | 0.9679 | 0.9732 | 0.9872 | 0.9884 | 0.9891 | 0.9896 | 0.9894 |
| **PSPNet** | 0.6574 | 0.7161 | 0.7221 | 0.7385 | 0.7659 | 0.7722 | 0.7834 | 0.7916 | 0.9526 | 0.9649 | 0.9606 | 0.9792 | 0.9802 | 0.9819 | 0.9834 | 0.9847 |
| **Fast-SCNN** | 0.1905 | 0.2507 | 0.4026 | 0.4892 | 0.4995 | 0.6416 | 0.6247 | 0.6547 | **0.9903** | **0.9907** | **0.9827** | 0.9808 | 0.9833 | 0.9794 | 0.9810 | 0.9807 |
| **TGANet** | 0.4753 | 0.5527 | 0.4993 | 0.6258 | 0.6096 | 0.6978 | 0.7058 | 0.6880 | 0.9408 | 0.9271 | 0.9424 | 0.9828 | 0.9866 | 0.9867 | 0.9871 | 0.9867 |
| **SegFormer** | 0.4267 | 0.5781 | 0.4013 | 0.6596 | 0.3907 | 0.6501 | 0.4442 | 0.6342 | 0.9160 | 0.9275 | 0.9255 | 0.9723 | **0.9897** | 0.9836 | **0.9912** | 0.9856 |
| **Unet++** | 0.4771 | 0.5027 | 0.6257 | 0.6391 | 0.7360 | 0.7334 | 0.9369 | 0.7788 | 0.8757 | 0.8885 | 0.8773 | 0.9761 | 0.9760 | 0.9822 | 0.7232 | 0.9817 |
| **autoSAM** | 0.6093 | 0.6603 | 0.5671 | 0.5575 | 0.5373 | 0.6481 | 0.6476 | 0.6600 | 0.9235 | 0.9199 | 0.9306 | 0.9811 | 0.9836 | 0.9845 | 0.9814 | 0.9839 |
| **Mamba-Unet** | 0.5819 | 0.6707 | 0.6763 | 0.6493 | 0.6761 | 0.6763 | 0.7060 | 0.7329 | 0.9567 | 0.9668 | 0.9692 | 0.9780 | 0.9798 | 0.9784 | 0.9771 | 0.9810 |
| **nnSAM(FCN)** | **0.6915** | 0.7439 | 0.6758 | 0.7633 | 0.7472 | 0.7993 | 0.8085 | 0.7970 | 0.9359 | 0.9533 | 0.9654 | 0.9857 | 0.9884 | **0.9892** | 0.9888 | 0.9895 |
| **proposed(FCN)** | 0.6869 | 0.7578 | 0.7003 | 0.8013 | **0.8112** | **0.8401** | **0.8454** | 0.8282 | 0.9492 | 0.9585 | 0.9680 | 0.9816 | 0.9852 | 0.9855 | 0.9857 | 0.9868 |
| **nnSAM(deep)** | 0.5976 | 0.6871 | 0.6995 | 0.7622 | 0.7898 | 0.7861 | 0.7981 | 0.8052 | 0.9638 | 0.9756 | 0.9747 | 0.9861 | 0.9874 | 0.9891 | 0.9896 | 0.9893 |
| **proposed(deep)** | 0.6829 | **0.7793** | **0.7740** | **0.8058** | 0.8007 | 0.8070 | 0.8226 | **0.8312** | 0.9566 | 0.9653 | 0.9682 | 0.9820 | 0.9509 | 0.9876 | 0.9880 | 0.9872 |

**Table S4. Comparison results of different methods with few-shot data with sensitivity and specificity.**

| **Method** | **Metrics** | | | | | | | | | | | | | | | |
|---|---|---|---|---|---|---|---|---|---|---|---|---|---|---|---|---|
| | **AUC** | | | | | | | | **AUPR** | | | | | | | |
| | **training sample size(n)** | | | | | | | | | | | | | | | |
| | 1 | 2 | 4 | 6 | 8 | 12 | 16 | 20 | 1 | 2 | 4 | 6 | 8 | 12 | 16 | 20 |
| **FCN** | 0.8798 | 0.9268 | 0.8928 | 0.9211 | 0.9126 | 0.9308 | 0.9336 | 0.9273 | 0.5112 | 0.6513 | 0.6101 | 0.7708 | 0.7834 | 0.8283 | 0.8409 | 0.8313 |
| **deeplabv3** | 0.8495 | 0.9123 | 0.8956 | 0.9235 | 0.9128 | 0.9386 | 0.9399 | 0.9469 | 0.5633 | 0.6648 | 0.6724 | 0.7706 | 0.7732 | 0.8310 | 0.8423 | 0.8519 |
| **PSPNet** | **0.9276** | 0.9328 | 0.9276 | 0.9197 | 0.9599 | 0.9325 | 0.9429 | 0.9433 | 0.5242 | 0.5880 | 0.5814 | 0.7116 | 0.7753 | 0.7701 | 0.7963 | 0.8121 |
| **Fast-SCNN** | 0.9052 | 0.9203 | 0.9312 | 0.8517 | 0.8565 | 0.8739 | 0.8778 | 0.8863 | 0.4320 | 0.5107 | 0.5480 | 0.5501 | 0.5731 | 0.6384 | 0.6476 | 0.6828 |
| **TGANet** | 0.7676 | 0.8438 | 0.7404 | 0.8049 | 0.7778 | 0.8212 | 0.8207 | 0.8354 | 0.3707 | 0.4333 | 0.4484 | 0.6102 | 0.6164 | 0.6752 | 0.6818 | 0.6877 |
| **SegFormer** | 0.8284 | 0.9122 | 0.8159 | 0.9587 | 0.7587 | 0.9647 | 0.7789 | 0.9679 | 0.2887 | 0.4161 | 0.2822 | 0.6302 | 0.4878 | 0.7177 | 0.5420 | 0.7361 |
| **Unet++** | 0.6397 | 0.7955 | 0.8386 | 0.8931 | 0.9311 | 0.9266 | 0.7089 | 0.9252 | 0.2500 | 0.2718 | 0.3540 | 0.6340 | 0.6978 | 0.7306 | 0.9847 | 0.7410 |
| **autoSAM** | 0.8179 | 0.9305 | 0.9079 | 0.9369 | 0.9132 | 0.9273 | 0.9461 | 0.9478 | 0.3325 | 0.5207 | 0.4992 | 0.6310 | 0.6240 | 0.6719 | 0.6875 | 0.6948 |
| **Mamba-Unet** | 0.8660 | 0.8902 | 0.8844 | 0.8602 | 0.8643 | 0.8658 | 0.8960 | 0.8878 | 0.4047 | 0.5011 | 0.5175 | 0.5470 | 0.5752 | 0.5617 | 0.5800 | 0.6307 |
| **nnSAM(FCN)** | 0.9031 | 0.9290 | 0.9277 | **0.9538** | 0.9583 | 0.9579 | 0.9592 | 0.9430 | 0.5810 | 0.6681 | 0.6563 | **0.8126** | **0.8377** | **0.8600** | **0.8639** | 0.8432 |
| **proposed(FCN)** | 0.8757 | 0.9119 | 0.8948 | 0.9497 | **0.9606** | **0.9749** | **0.9734** | **0.9727** | 0.5065 | 0.5923 | 0.5894 | 0.7623 | 0.8051 | 0.8423 | 0.8513 | 0.8434 |
| **nnSAM(deep)** | 0.9003 | 0.9310 | 0.9363 | 0.9481 | 0.9386 | 0.9499 | 0.9460 | 0.9533 | **0.5939** | **0.6864** | **0.6848** | 0.7921 | 0.8014 | 0.8409 | 0.8462 | **0.8565** |
| **proposed(deep)** | 0.9128 | **0.9408** | **0.9401** | 0.9461 | 0.9509 | **0.9749** | 0.9692 | 0.9704 | 0.5625 | 0.6568 | 0.6339 | 0.7569 | 0.7818 | 0.8428 | 0.8496 | 0.8444 |

**Table S5. Comparison results of different methods with few-shot data with AUC and AUPR.**

| Method | metrics | training sample n=1 | | | |
|---|---|---|---|---|---|
| | | Endocardium | Epicardium | Left Atrium wall | Average |
| **FCN** | **DICE** | 0.5820 | 0.4327 | 0.4311 | 0.4820 |
| **deeplabv3** | **DICE** | **0.7112** | 0.4248 | 0.4112 | 0.5157 |
| **PSPNet** | **DICE** | 0.6523 | 0.4524 | 0.4877 | 0.5308 |
| **Fast-SCNN** | **DICE** | 0.3464 | 0.394 | 0.0073 | 0.2310 |
| **TGANet** | **DICE** | 0.5105 | 0.4760 | 0.0296 | 0.3387 |
| **SegFormer** | **DICE** | 0.4228 | 0.3503 | 0.0181 | 0.2637 |
| **Unet++** | **DICE** | 0.3776 | 0.3347 | 0.0336 | 0.2486 |
| **autoSAM** | **DICE** | 0.5472 | 0.4461 | 0.3802 | 0.4578 |
| **Mamba-Unet** | **DICE** | 0.6449 | 0.4258 | 0.4414 | 0.5040 |
| **nnSAM(FCN)** | **DICE** | 0.5748 | 0.5106 | 0.4408 | 0.5087 |
| **proposed(FCN)** | **DICE** | 0.6317 | 0.5069 | 0.4872 | 0.5419 |
| **nnSAM(deep)** | **DICE** | 0.6637 | 0.4908 | 0.4425 | 0.5323 |
| **proposed(deep)** | **DICE** | 0.6829 | **0.5372** | **0.5073** | **0.5758** |
| **FCN** | **HD** | 37.1553 | 29.8310 | 33.6033 | 33.5299 |
| **deeplabv3** | **HD** | **18.0074** | 35.6166 | 25.2696 | 26.2979 |
| **PSPNet** | **HD** | 22.0745 | 21.9019 | 25.3259 | 23.1007 |
| **Fast-SCNN** | **HD** | 34.9323 | 35.3507 | 35.2037 | 35.1622 |
| **TGANet** | **HD** | 61.9677 | 40.5671 | 31.3079 | 44.6142 |
| **SegFormer** | **HD** | 78.2713 | 34.7020 | 37.5240 | 50.1658 |
| **Unet++** | **HD** | 81.4758 | 66.8835 | 52.4296 | 66.9296 |
| **autoSAM** | **HD** | 68.8390 | 25.3871 | 32.3451 | 42.1904 |
| **Mamba-Unet** | **HD** | 22.4172 | 22.3985 | 25.9283 | 23.5813 |
| **nnSAM(FCN)** | **HD** | 39.1693 | 21.6474 | 37.1970 | 32.6712 |
| **proposed(FCN)** | **HD** | 28.1233 | **19.8188** | 28.6292 | 25.5238 |
| **nnSAM(deep)** | **HD** | 25.8006 | 21.9937 | 24.9907 | 24.2617 |
| **proposed(deep)** | **HD** | 19.9366 | 20.5487 | **21.8444** | **20.7766** |

**Table S6. Comparison results of different methods with one train sample with DICE and HD.**

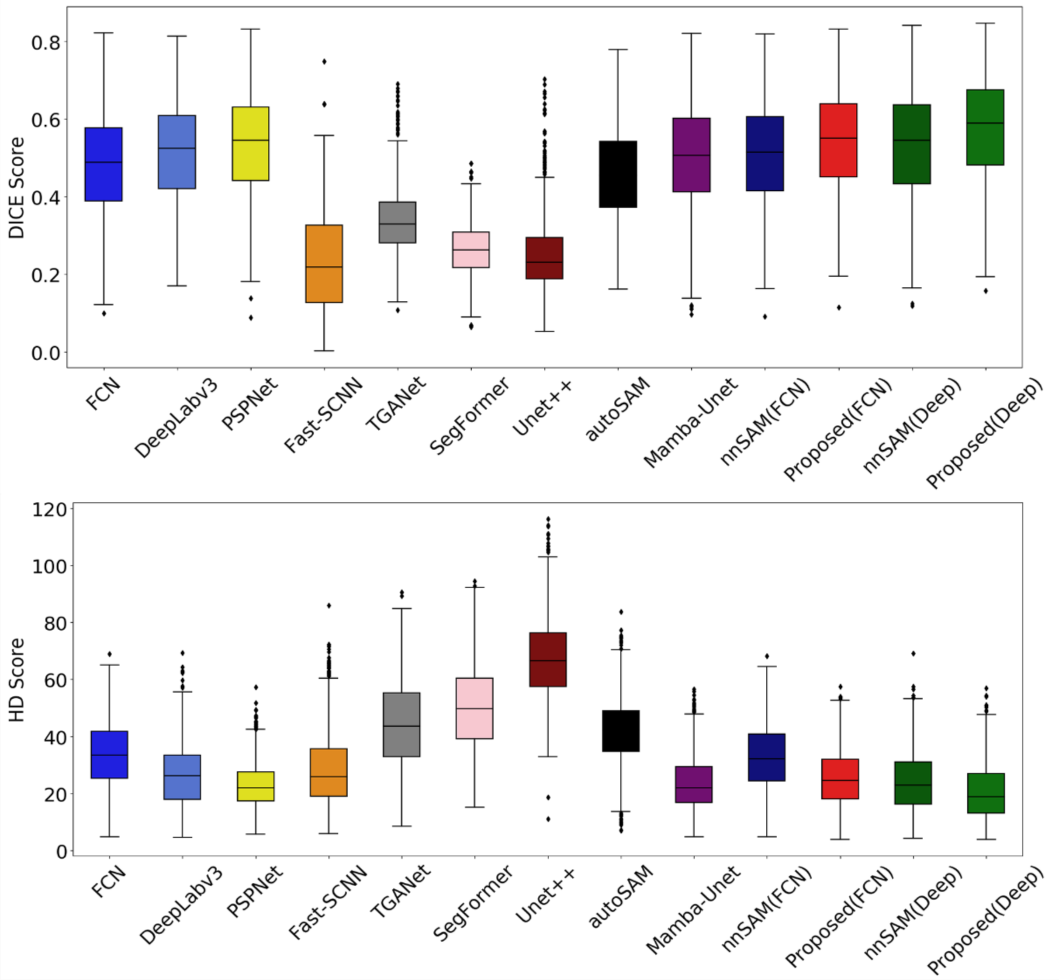


**Figure S2. | Comparison results of different methods with one sample.**

| Method | metrics | Endocardium | Epicardium | Left Atrium wall | Average |
|---|---|---|---|---|---|
| **FCN** | **DICE** | 0.9293 | 0.8608 | 0.8957 | 0.8953 |
| **deeplabv3** | **DICE** | 0.9288 | 0.8612 | 0.8952 | 0.8951 |
| **PSPNet** | **DICE** | 0.9220 | 0.8543 | 0.8830 | 0.8864 |
| **Fast-SCNN** | **DICE** | 0.8975 | 0.8132 | 0.8445 | 0.8517 |
| **TGANet** | **DICE** | 0.9160 | 0.8354 | 0.8706 | 0.8741 |
| **SegFormer** | **DICE** | 0.8796 | 0.7849 | 0.0000 | 0.5551 |
| **Unet++** | **DICE** | 0.9226 | 0.8537 | 0.8785 | 0.8849 |
| **autoSAM** | **DICE** | 0.9044 | 0.8226 | 0.8476 | 0.8582 |
| **Mamba-Unet** | **DICE** | 0.9060 | 0.8278 | 0.8695 | 0.8678 |
| **nnSAM(FCN)** | **DICE** | 0.9300 | 0.8614 | 0.8958 | 0.8957 |
| **Proposed(FCN)** | **DICE** | 0.9299 | 0.8624 | 0.8963 | 0.8962 |
| **nnSAM(deeplab)** | **DICE** | 0.9294 | 0.8611 | **0.8966** | 0.8957 |
| **Proposed(deeplab)** | **DICE** | **0.9304** | **0.8631** | 0.8953 | **0.8963** |

**Table S7. Results of different methods in comparison analysis with DICE**

| Method | metrics | Endocardium | Epicardium | Left Atrium wall | Average |
|---|---|---|---|---|---|
| **FCN** | **HD** | 1.4259 | 2.9085 | 2.8840 | 2.4061 |
| **deeplabv3** | **HD** | 1.4926 | 2.9490 | 2.7590 | 2.4002 |
| **PSPNet** | **HD** | 1.7128 | 3.0659 | 3.1702 | 2.6497 |
| **Fast-SCNN** | **HD** | 3.0625 | 4.6298 | 4.9506 | 4.2143 |
| **TGANet** | **HD** | 2.2168 | 3.9512 | 4.0042 | 3.3908 |
| **SegFormer** | **HD** | 3.8521 | 5.5691 | 10.6234 | 6.6815 |
| **Unet++** | **HD** | 1.8188 | 3.3839 | 3.7397 | 2.9808 |
| **autoSAM** | **HD** | 2.7635 | 4.2691 | 5.1145 | 4.0491 |
| **Mamba-Unet** | **HD** | 2.6295 | 4.0745 | 4.0095 | 3.5711 |
| **nnSAM(FCN)** | **HD** | 1.4157 | 2.9323 | 2.7728 | 2.3736 |
| **Proposed(FCN)** | **HD** | 1.4144 | 2.8919 | 2.8212 | 2.3759 |
| **nnSAM(deeplab)** | **HD** | 1.3983 | 2.8846 | **2.6668** | 2.3166 |
| **Proposed(deeplab)** | **HD** | **1.3205** | **2.8371** | 2.7179 | **2.2919** |

**Table S8. Results of different methods in comparison analysis with HD**

As the quantity of training data reached a threshold, a convergence of metrics was observed across methods. Specifically, classical methods like FCN and DeepLabV3 exhibited competitive metrics, outperforming contemporary state-of-the-art approaches including autoSAM integrated with SAM under the same training strategy. Integrating SAM further improved metrics for FCN (89.53 to 89.57) and DeepLabV3 (89.51 to 89.57). However, the proposed approach using SAM as the primary model along with an auxiliary guiding model achieved peak performance, with the highest metrics recorded (89.57 to 89.63) (Table S7).

The proposed method showed improvements over baselines across segmentation categories. Comparable networks also outperformed nnSAM, though less substantially. Epicardium segmentation saw the greatest enhancement (86.11 to 86.31), while minimal change or regression occurred for Left Atrium wall (89.66 to 89.53). Additionally, the epicardium had the lowest global DICE values. Both epicardium and left atrium wall performed worse on the HD contour metric versus endocardium (Table S8), indicating difficulty perfectly segmenting small, sensitive outer walls, especially contour alignment. Further efforts to improve accuracy remain necessary.

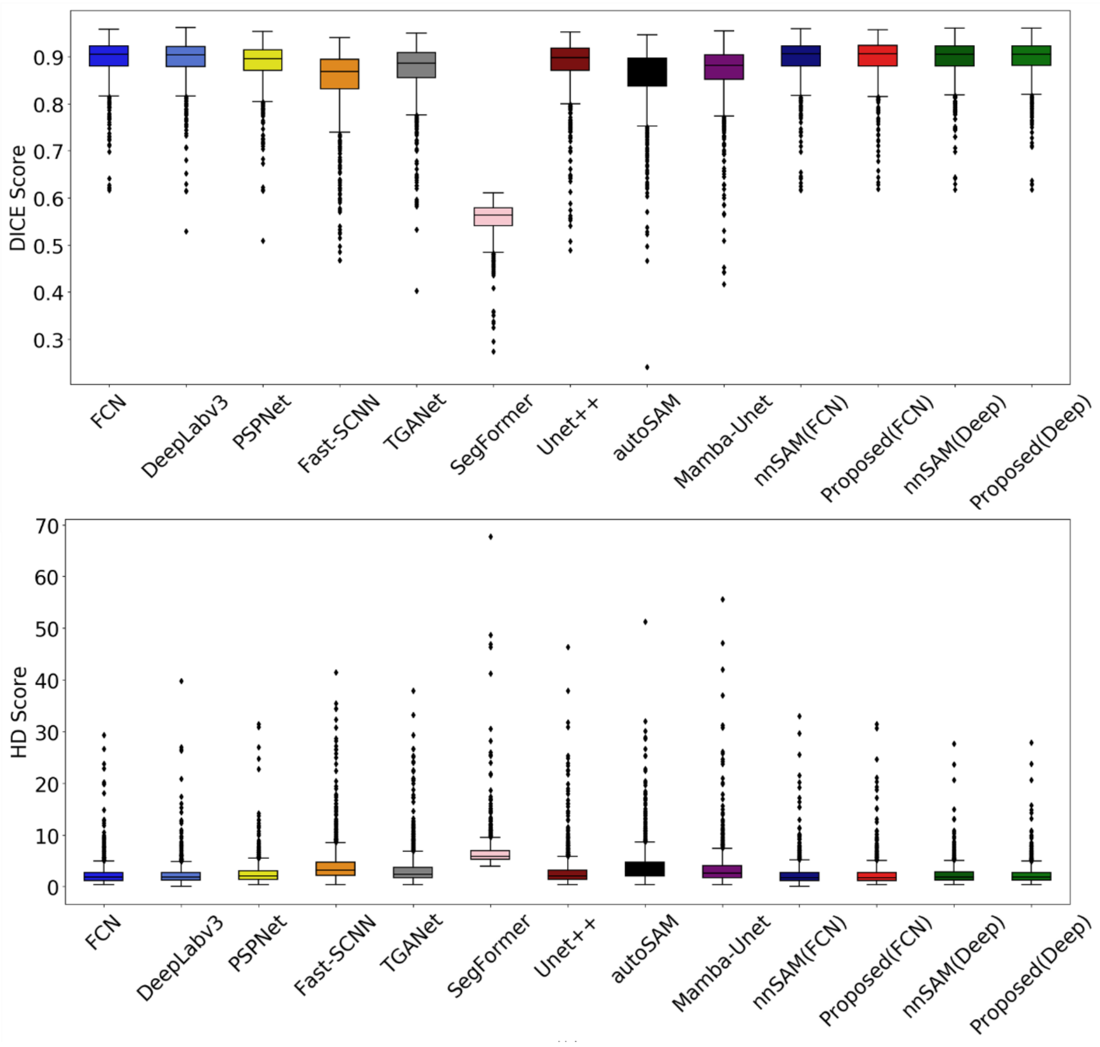


**Figure S3 | Comparison results of different methods with full training data**

Overall, the proposed method's metrics were relatively stable (Figure S3). Although DeepLabV3 and nnSAM (Deep) had similar means to proposed (Deep), metric stability differed noticeably. In terms of accuracy and stability, the proposed approach achieved the best results, signaling the potential of retaining more experience to further optimize framework performance.

# 4. Comparison of performance among different methods in REFUGE

| Method | Metrics | | | | | | | | | | | | | | | |
|---|---|---|---|---|---|---|---|---|---|---|---|---|---|---|---|---|
| | SEN | | | | | | | | SPEC | | | | | | | |
| | training sample size(n) | | | | | | | | | | | | | | | |
| | 1 | 2 | 4 | 6 | 8 | 12 | 16 | 20 | 1 | 2 | 4 | 6 | 8 | 12 | 16 | 20 |
| **FCN** | 0.3750 | 0.6095 | 0.7414 | 0.7897 | 0.7735 | 0.8646 | 0.8657 | 0.8617 | 0.9998 | 0.9993 | 0.9992 | 0.9993 | 0.9994 | 0.9992 | 0.9992 | 0.9994 |
| **deeplabv3** | 0.4775 | 0.6239 | 0.7986 | 0.7801 | 0.8062 | 0.8627 | 0.8616 | **0.8588** | **0.9989** | **0.9996** | **0.9991** | **0.9993** | **0.9994** | **0.9993** | **0.9994** | **0.9994** |
| **PSPNet** | 0.2314 | 0.4830 | 0.6587 | 0.7809 | 0.8073 | 0.8972 | 0.8790 | 0.9022 | 0.9991 | 0.9980 | 0.9985 | 0.9982 | 0.9986 | 0.9983 | 0.9988 | 0.9985 |
| **Fast-SCNN** | 0.3166 | 0.4703 | 0.6255 | 0.6198 | 0.6353 | 0.7551 | 0.7434 | 0.7781 | 0.9981 | 0.9994 | 0.9989 | 0.9991 | 0.9992 | 0.9990 | 0.9990 | 0.9985 |
| **TGANet** | 0.5148 | 0.6477 | 0.7217 | 0.7647 | 0.7414 | 0.8191 | 0.8029 | 0.7889 | 0.9969 | 0.9966 | 0.9880 | 0.9895 | 0.9983 | 0.9985 | 0.9981 | 0.9987 |
| **SegFormer** | 0.6589 | 0.5934 | 0.6825 | 0.7272 | 0.7499 | 0.7868 | 0.7310 | 0.7347 | 0.9874 | 0.9985 | 0.9977 | 0.9987 | 0.9985 | 0.9975 | 0.9984 | 0.9984 |
| **Unet++** | 0.6921 | 0.7064 | 0.8077 | 0.8298 | 0.8441 | 0.8845 | 0.8657 | 0.8714 | 0.9819 | 0.7424 | 0.9972 | 0.9977 | 0.9950 | 0.9961 | 0.9973 | 0.9982 |
| **autoSAM** | 0.3868 | 0.4115 | 0.7435 | 0.7538 | 0.7925 | 0.8105 | 0.7704 | 0.8099 | 0.9986 | 0.9980 | 0.9974 | 0.9984 | 0.9980 | 0.9983 | 0.9989 | 0.9983 |
| **Mamba-Unet** | 0.2197 | 0.3061 | 0.3351 | 0.6922 | 0.6827 | 0.8025 | 0.7014 | 0.7561 | 0.9789 | 0.9966 | 0.9955 | 0.9926 | 0.9983 | 0.9964 | 0.9944 | 0.9987 |
| **nnSAM(FCN)** | 0.5153 | 0.7517 | 0.7682 | 0.7787 | 0.8260 | 0.8610 | 0.8407 | 0.8522 | 0.9993 | 0.9992 | 0.9992 | 0.9993 | 0.9992 | 0.9993 | 0.9994 | 0.9994 |
| **proposed(FCN)** | 0.7560 | 0.7761 | 0.8571 | 0.8480 | 0.8247 | **0.8700** | 0.8655 | 0.8641 | 0.9987 | 0.9992 | 0.9989 | 0.9991 | 0.9992 | 0.9992 | 0.9992 | 0.9994 |
| **nnSAM(deep)** | 0.5418 | 0.6274 | 0.7775 | 0.7912 | 0.8214 | 0.8667 | 0.8508 | 0.8513 | 0.9992 | 0.9992 | 0.9990 | 0.9991 | 0.9992 | 0.9992 | 0.9993 | 0.9995 |
| **proposed(deep)** | 0.6525 | **0.6927** | 0.8254 | **0.9355** | 0.8785 | 0.8711 | 0.9007 | 0.8658 | 0.9992 | 0.9992 | 0.9990 | 0.9989 | 0.9988 | 0.9991 | 0.9989 | 0.9994 |

**Tabel S9. Comparison results of different methods with few-shot data with sensitivity and specificity.**

| **Method** | **Metrics** | | | | | | | | | | | | | | | |
|---|---|---|---|---|---|---|---|---|---|---|---|---|---|---|---|---|
| | **AUC** | | | | | | | | **AUPR** | | | | | | | |
| | **training sample size(n)** | | | | | | | | | | | | | | | |
| | 1 | 2 | 4 | 6 | 8 | 12 | 16 | 20 | 1 | 2 | 4 | 6 | 8 | 12 | 16 | 20 |
| **FCN** | 0.7336 | 0.8694 | 0.9519 | 0.9613 | 0.9637 | 0.9757 | 0.9758 | 0.9748 | 0.6097 | 0.7684 | 0.8813 | 0.9041 | 0.9093 | 0.9410 | 0.9406 | 0.9483 |
| **deeplabv3** | 0.9315 | 0.9799 | 0.9869 | 0.9889 | 0.9873 | 0.9887 | 0.9879 | **0.9853** | **0.7118** | **0.8866** | **0.9279** | **0.9488** | **0.9505** | **0.9633** | **0.9613** | **0.9628** |
| **PSPNet** | 0.7954 | 0.9765 | 0.9779 | 0.9800 | 0.9753 | 0.9909 | 0.9925 | 0.9928 | 0.3557 | 0.6177 | 0.7858 | 0.8653 | 0.8947 | 0.9395 | 0.9460 | 0.9511 |
| **Fast-SCNN** | 0.8330 | 0.9478 | 0.9662 | 0.9355 | 0.9625 | 0.9560 | 0.9628 | 0.9738 | 0.4307 | 0.7152 | 0.8032 | 0.7944 | 0.8215 | 0.8816 | 0.8900 | 0.90051 |
| **TGANet** | 0.6680 | 0.7521 | 0.8071 | 0.8407 | 0.8217 | 0.9038 | 0.8948 | 0.8822 | 0.5858 | 0.6736 | 0.7162 | 0.7716 | 0.7630 | 0.8466 | 0.8332 | 0.8307 |
| **SegFormer** | 0.9823 | 0.9960 | 0.9961 | 0.9958 | 0.9958 | 0.9974 | 0.9977 | 0.9979 | 0.7246 | 0.8344 | 0.8674 | 0.8839 | 0.8840 | 0.9001 | 0.9007 | 0.9071 |
| **Unet++** | 0.7522 | 0.8066 | 0.8906 | 0.8992 | 0.8852 | 0.9261 | 0.9124 | 0.9249 | 0.6154 | 0.7004 | 0.7913 | 0.8173 | 0.8206 | 0.8579 | 0.8605 | 0.8761 |
| **autoSAM** | 0.9798 | 0.9698 | 0.9958 | 0.9970 | 0.9976 | 0.9981 | 0.9989 | 0.9987 | 0.7153 | 0.7090 | 0.8878 | 0.9012 | 0.9198 | 0.9297 | 0.9395 | 0.9389 |
| **Mamba-Unet** | 0.6611 | 0.7399 | 0.7811 | 0.9601 | 0.8852 | 0.9959 | 0.9775 | 0.9696 | 0.2655 | 0.3300 | 0.3504 | 0.6573 | 0.8206 | 0.8493 | 0.7683 | 0.8149 |
| **nnSAM(FCN)** | 0.9104 | 0.9848 | 0.9724 | 0.9689 | 0.9894 | 0.9814 | 0.9790 | 0.9770 | 0.6761 | 0.8720 | 0.8974 | 0.8866 | 0.9402 | 0.9411 | 0.9482 | 0.9490 |
| **proposed(FCN)** | 0.9825 | 0.9867 | 0.9883 | 0.9935 | 0.9849 | **0.9880** | 0.9811 | 0.9865 | 0.8456 | 0.8905 | 0.9397 | 0.9537 | 0.9367 | 0.9599 | 0.9556 | 0.9604 |
| **nnSAM(deep)** | 0.9417 | 0.9703 | 0.9874 | 0.9858 | 0.9921 | 0.9891 | 0.9859 | 0.9877 | 0.7227 | 0.8319 | 0.9105 | 0.9364 | 0.9520 | 0.9630 | 0.9592 | 0.9624 |
| **proposed(deep)** | 0.9916 | **0.9931** | 0.9939 | **0.9971** | 0.9960 | 0.9916 | 0.9954 | 0.9892 | 0.8589 | 0.8937 | 0.9284 | 0.9497 | 0.9557 | 0.9619 | 0.9672 | 0.9647 |

**Tabel S10. Comparison results of different methods with few-shot data with AUC and AUPR.**

| Method | metrics | training sample n=1 | | |
|---|---|---|---|---|
| | | Optic Cup | Optic Disc | Mean |
| **FCN** | DICE | 0.3602 | 0.5978 | 0.4790 |
| **deeplabv3** | DICE | 0.4929 | 0.6386 | 0.5657 |
| **PSPNet** | DICE | 0.1945 | 0.3959 | 0.2952 |
| **Fast-SCNN** | DICE | 0.2342 | 0.4603 | 0.3473 |
| **TGANet** | DICE | 0.4287 | 0.7212 | 0.5750 |
| **SegFormer** | DICE | 0.5856 | 0.6173 | 0.6014 |
| **Unet++** | DICE | 0.4040 | 0.6350 | 0.5195 |
| **autoSAM** | DICE | 0.3247 | 0.6476 | 0.4861 |
| **Mamba-Unet** | DICE | 0.2501 | 0.2632 | 0.2567 |
| **nnSAM(FCN)** | DICE | 0.5050 | 0.6696 | 0.5873 |
| **proposed(FCN)** | DICE | **0.6293** | **0.7794** | **0.7044** |
| **nnSAM(deep)** | DICE | 0.5206 | 0.6268 | 0.5737 |
| **proposed(deep)** | DICE | 0.5897 | 0.6809 | 0.6353 |
| **FCN** | HD | 23.97 | 16.16 | 20.06 |
| **deeplabv3** | HD | 17.80 | 20.57 | 19.19 |
| **PSPNet** | HD | 45.25 | 28.10 | 36.68 |
| **Fast-SCNN** | HD | 46.51 | 44.41 | 45.46 |
| **TGANet** | HD | 29.00 | 23.52 | 26.26 |
| **SegFormer** | HD | 20.80 | 39.47 | 30.13 |
| **Unet++** | HD | 43.39 | 39.34 | 41.36 |
| **autoSAM** | HD | 32.92 | 11.88 | 22.40 |
| **Mamba-Unet** | HD | 29.19 | 53.76 | 41.47 |
| **nnSAM(FCN)** | HD | 14.67 | 14.49 | 14.58 |
| **proposed(FCN)** | HD | **10.50** | **5.62** | **8.06** |
| **nnSAM(deep)** | HD | 22.51 | 23.02 | 22.76 |
| **proposed(deep)** | HD | 16.20 | 11.67 | 13.93 |

**Table S11. Comparison results of different methods with one train sample with DICE and HD in REFUGE dataset.**

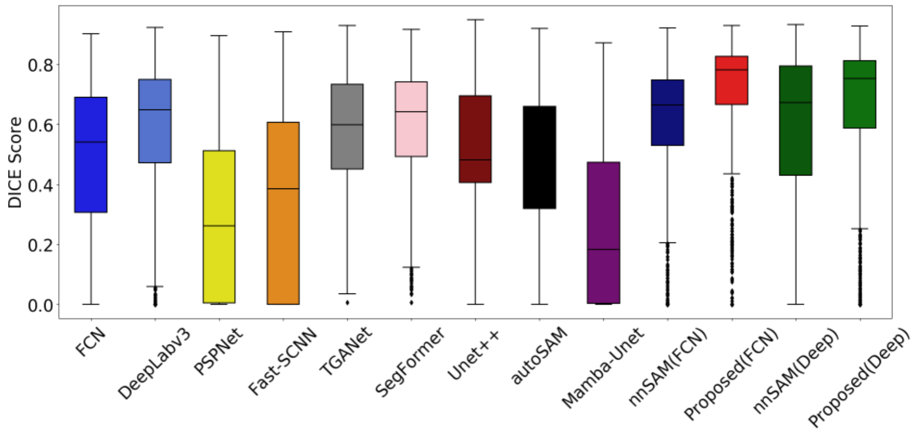


**Figure S4. | Comparison results of different methods with one sample with DICE in REFUGE dataset.**

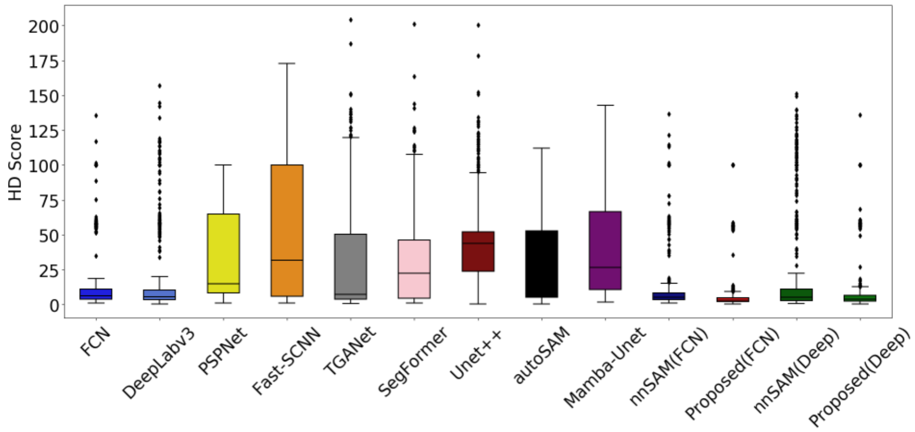


**Figure S5. | Comparison results of different methods with one sample with HD in REFUGE dataset.**

# 5. Comparison of computational efficiency among different methods

| Method | Parameter | FLOPs | Speed |
| --- | --- | --- | --- |
| FCN | 32.9M | 3.48*e10 | 107 it/s |
| DeepLabV3 | 39.6M | 4.11*e10 | 103 it/s |
| Unet++ | 9.2M | 3.48*e10 | 125 it/s |
| PSPNet | 53.6M | 0.72*e10 | 102 it/s |
| Fast-SCNN | 1.1M | 0.02*e10 | 133 it/s |
| SegFormer | 7.7M | 0.33*e10 | 104 it/s |
| TGANet | 19.8M | 3.55*e10 | 65 it/s |
| AutoSAM | 93.5M | 49.18*e10 | 30 it/s |
| Mamba-Unet | 27.4M | 2.79*e10 | 12 it/s |
| nnSAM(FCN) | 0.3G | 9.04e*10 | 37 it/s |
| Proposed(FCN) (FACM ON) | 0.4G | 11.00*e10 | 34 it/s |
| Proposed(FCN)(FACM OFF) | 0.4G | 11.00*e10 | 34 it/s |
| nnSAM(deep) | 0.4G | 11.25*e10 | 38 it/s |
| Proposed(deep) (FACM ON) | 0.4G | 11.57*e10 | 35 it/s |
| Proposed(FCN) (FACM OFF) | 0.4G | 11.57*e10 | 35 it/s |

**Table S12. Computational efficiency of different methods and FACM**

## 6. Comparison of performance among different methods under data augmentation scenarios

| Hyper parameters | Data augmentation | |
|---|---|---|
| | No data Aug | Data Aug |
| **Image size** | 256*256 | 256*256 |
| **Random rotate** | Random rotate ±10° | Random rotate ±10° |
| **Random translate** | / | Dx: -25~25<br>Dy: -25~25 |
| **Random scale** | / | Random scale 0.5~1.25 |

**Table S13. The specific settings parameters for data augmentation.**

| Method | metrics | training sample n=1 CAMUS | | | |
|---|---|---|---|---|---|
| | | Endocardium | Epicardium | Left Atrium wall | Average |
| **FCN** | DICE | 0.6069 | 0.5885 | 0.4196 | 0.5383 |
| **deeplabv3** | DICE | 0.6911 | 0.6041 | 0.3278 | 0.5410 |
| **PSPNet** | DICE | 0.6304 | 0.5128 | 0.4567 | 0.5333 |
| **Fast-SCNN** | DICE | 0.5001 | 0.3779 | 0.3598 | 0.4126 |
| **TGANet** | DICE | 0.4802 | 0.5149 | 0.1192 | 0.3714 |
| **SegFormer** | DICE | 0.3801 | 0.4066 | 0.1710 | 0.3192 |
| **Unet++** | DICE | 0.3860 | 0.3686 | 0.0396 | 0.2647 |
| **autoSAM** | DICE | 0.4933 | 0.4606 | 0.0301 | 0.3280 |
| **Mamba-Unet** | DICE | 0.6835 | 0.5024 | 0.4625 | 0.5495 |
| **nnSAM(FCN)** | DICE | 0.6705 | 0.5484 | 0.4102 | 0.5430 |
| **proposed(FCN)** | DICE | 0.7388 | 0.5661 | 0.4589 | 0.5863 |
| **nnSAM(deep)** | DICE | 0.7388 | 0.5538 | 0.4826 | 0.5917 |
| **proposed(deep)** | DICE | 0.6988 | 0.5927 | 0.5492 | 0.6136 |
| **FCN** | HD | 42.00 | 23.15 | 35.96 | 33.70 |
| **deeplabv3** | HD | 28.92 | 19.93 | 33.04 | 27.97 |
| **PSPNet** | HD | 42.61 | 37.11 | 38.42 | 39.38 |
| **Fast-SCNN** | HD | 57.90 | 46.08 | 35.44 | 46.47 |
| **TGANet** | HD | 76.89 | 50.42 | 43.09 | 56.80 |
| **SegFormer** | HD | 80.25 | 60.99 | 47.14 | 62.80 |
| **Unet++** | HD | 80.9229 | 75.3619 | 55.9351 | 70.74 |
| **autoSAM** | HD | 69.13 | 69.55 | 36.65 | 58.45 |
| **Mamba-Unet** | HD | 19.85 | 20.79 | 24.84 | 21.53 |
| **nnSAM(FCN)** | HD | 31.37 | 25.35 | 50.62 | 35.78 |
| **proposed(FCN)** | HD | 18.33 | 22.79 | 35.27 | 25.46 |
| **nnSAM(deep)** | HD | 22.81 | 24.53 | 29.22 | 25.52 |
| **proposed(deep)** | HD | 10.38 | 11.02 | 14.47 | 11.96 |

**Table S14. Comparison results of different methods with one train sample with DICE and HD in CAMUS dataset with data augmentation.**

| Method | metrics | training sample n=1 CAMUS | |
|---|---|---|---|
| | | No data Aug | Data Aug |
| **FCN** | DICE | 0.4819 | **0.5383** |
| **deeplabv3** | DICE | 0.5157 | **0.5410** |
| **PSPNet** | DICE | 0.5308 | **0.5333** |
| **Fast-SCNN** | DICE | 0.2311 | **0.4126** |
| **TGANet** | DICE | 0.3387 | **0.3714** |
| **SegFormer** | DICE | 0.2637 | **0.3192** |
| **Unet++** | DICE | 0.2486 | **0.2647** |
| **autoSAM** | DICE | 0.4482 | **0.3280** |
| **Mamba-Unet** | DICE | 0.5040 | **0.5495** |
| **nnSAM(FCN)** | DICE | 0.5087 | **0.5430** |
| **proposed(FCN)** | DICE | 0.5419 | **0.5863** |
| **nnSAM(deep)** | DICE | 0.5323 | **0.5917** |
| **proposed(deep)** | DICE | 0.5758 | **0.6136** |
| **FCN** | HD | **33.53** | 33.70 |
| **deeplabv3** | HD | **26.29** | 27.97 |
| **PSPNet** | HD | **23.10** | 39.38 |
| **Fast-SCNN** | HD | **35.16** | 46.47 |
| **TGANet** | HD | **44.61** | 56.80 |
| **SegFormer** | HD | **50.16** | 62.80 |
| **Unet++** | HD | **66.93** | 70.74 |
| **autoSAM** | HD | **42.19** | 58.45 |
| **Mamba-Unet** | HD | **23.58** | 21.53 |
| **nnSAM(FCN)** | HD | **32.67** | 35.78 |
| **proposed(FCN)** | HD | 25.52 | **25.46** |
| **nnSAM(deep)** | HD | **24.26** | 25.52 |
| **proposed(deep)** | HD | 20.78 | **11.96** |

**Table S15. Comparison results of different data augmentation with one train sample with DICE and HD in CAMUS dataset with different methods.**

| Method | metrics | training sample n=1 REFUGE | | |
|---|---|---|---|---|
| | | Optic Cup | Optic Disc | Mean |
| **FCN** | DICE | 0.5666 | **0.7367** | 0.6516 |
| **deeplabv3** | DICE | 0.5091 | **0.7367** | 0.6229 |
| **PSPNet** | DICE | 0.6556 | 0.6678 | 0.6617 |
| **Fast-SCNN** | DICE | 0.5367 | 0.6199 | 0.5783 |
| **TGANet** | DICE | 0.4678 | 0.7081 | 0.5880 |
| **SegFormer** | DICE | 0.5902 | 0.7050 | 0.6476 |
| **Unet++** | DICE | 0.4390 | 0.6520 | 0.5455 |
| **autoSAM** | DICE | 0.4012 | 0.6362 | 0.5187 |
| **Mamba-Unet** | DICE | 0.4251 | 0.4350 | 0.4300 |
| **nnSAM(FCN)** | DICE | 0.6037 | 0.7115 | 0.6576 |
| **proposed(FCN)** | DICE | 0.6940 | 0.7024 | 0.6982 |
| **nnSAM(deep)** | DICE | 0.5848 | 0.6836 | 0.6342 |
| **proposed(deep)** | DICE | **0.7140** | 0.7052 | **0.7096** |
| **FCN** | HD | 13.00 | 9.26 | 11.13 |
| **deeplabv3** | HD | 20.63 | 9.53 | 15.08 |
| **PSPNet** | HD | **6.01** | 7.17 | **6.59** |
| **Fast-SCNN** | HD | 12.05 | 18.96 | 15.51 |
| **TGANet** | HD | 33.39 | 36.98 | 35.19 |
| **SegFormer** | HD | 20.92 | 29.67 | 25.29 |
| **Unet++** | HD | 31.81 | 37.92 | 34.87 |
| **autoSAM** | HD | 21.43 | 18.11 | 19.77 |
| **Mamba-Unet** | HD | 58.46 | 70.49 | 64.48 |
| **nnSAM(FCN)** | HD | 14.33 | 13.92 | 14.12 |
| **proposed(FCN)** | HD | 9.31 | 7.74 | 8.53 |
| **nnSAM(deep)** | HD | 17.25 | 14.75 | 15.99 |
| **proposed(deep)** | HD | 7.44 | **6.52** | 7.13 |

**Table S16. Comparison results of different methods with one train sample with DICE and HD in REFUGE dataset with data augmentation.**

| Method | metrics | training sample n=1 REFUGE | |
|---|---|---|---|
| | | No data Aug | Data Aug |
| **FCN** | DICE | 0.4790 | **0.6516** |
| **deeplabv3** | DICE | 0.5657 | **0.6229** |
| **PSPNet** | DICE | 0.2952 | **0.6617** |
| **Fast-SCNN** | DICE | 0.3473 | **0.5783** |
| **TGANet** | DICE | 0.5750 | **0.5880** |
| **SegFormer** | DICE | 0.6014 | **0.6476** |
| **Unet++** | DICE | 0.5241 | **0.5455** |
| **autoSAM** | DICE | 0.4723 | **0.5187** |
| **Mamba-Unet** | DICE | 0.2567 | **0.4300** |
| **nnSAM(FCN)** | DICE | 0.6049 | **0.6576** |
| **proposed(FCN)** | DICE | **0.7141** | 0.6982 |
| **nnSAM(deep)** | DICE | **0.6347** | 0.6342 |
| **proposed(deep)** | DICE | 0.6725 | **0.7096** |
| **FCN** | HD | 20.0650 | **11.13** |
| **deeplabv3** | HD | 19.1857 | **15.08** |
| **PSPNet** | HD | 36.6751 | **6.59** |
| **Fast-SCNN** | HD | 45.4614 | **15.51** |
| **TGANet** | HD | **26.2602** | 35.19 |
| **SegFormer** | HD | 30.1322 | **25.29** |
| **Unet++** | HD | 41.3638 | **34.87** |
| **autoSAM** | HD | 22.5677 | **19.77** |
| **Mamba-Unet** | HD | **41.4738** | 64.48 |
| **nnSAM(FCN)** | HD | **13.0598** | 14.12 |
| **proposed(FCN)** | HD | **7.5007** | 8.53 |
| **nnSAM(deep)** | HD | **10.5907** | 15.99 |
| **proposed(deep)** | HD | 11.8223 | **7.13** |

**Table S17. Comparison results of different data augmentation with one train sample with DICE and HD in REFUGE dataset with different methods.**

# 7. Comparison of performance of automatic prompt and manual prompt

| Method | Metrics | | | | | | | | | | | | | | | |
|---|---|---|---|---|---|---|---|---|---|---|---|---|---|---|---|---|
| | DICE | | | | | | | | HD | | | | | | | |
| | training sample size(n) | | | | | | | | | | | | | | | |
| | 1 | 2 | 4 | 6 | 8 | 12 | 16 | 20 | 1 | 2 | 4 | 6 | 8 | 12 | 16 | 20 |
| **proposed(FCN)** | 0.5419 | 0.6164 | 0.6103 | 0.7570 | 0.7818 | 0.8060 | 0.8091 | 0.8052 | 25.5238 | 23.3281 | 19.4167 | 10.5465 | 8.7999 | 7.9875 | 7.1839 | 7.8991 |
| **proposed(deep)** | 0.5758 | 0.6584 | 0.6519 | 0.7599 | 0.7672 | 0.7973 | 0.8091 | 0.8104 | 20.7766 | 17.5505 | 16.7683 | 11.8957 | 10.3071 | 8.7514 | 7.8906 | 7.6765 |
| SAMMed2D 5 points | 0.4815 | | | | | | | | 18.3526 | | | | | | | |
| SAMMed2D 16 points | 0.5884 | | | | | | | | 11.2584 | | | | | | | |
| SAMMed2D 32 points | 0.6795 | | | | | | | | 6.9085 | | | | | | | |

**Table S18. Comparison results of proposed method based on automatic prompt and SAM Med2d based on manual points prompt in CAMUS dataset.**

| Method | Metrics | | | | | | | | | | | | | | | |
|---|---|---|---|---|---|---|---|---|---|---|---|---|---|---|---|---|
| | DICE | | | | | | | | HD | | | | | | | |
| | training sample size(n) | | | | | | | | | | | | | | | |
| | 1 | 2 | 4 | 6 | 8 | 12 | 16 | 20 | 1 | 2 | 4 | 6 | 8 | 12 | 16 | 20 |
| **proposed(FCN)** | 0.7141 | 0.7898 | 0.8427 | 0.8449 | 0.8432 | 0.8743 | 0.8773 | 0.8800 | 7.5007 | 4.6304 | 2.3837 | 2.3026 | 2.3543 | 1.7615 | 1.8195 | 1.5801 |
| **proposed(deep)** | 0.6725 | 0.7282 | 0.8075 | 0.8229 | 0.8329 | 0.8674 | 0.8741 | 0.8784 | 11.8223 | 7.5185 | 4.2160 | 3.3738 | 2.6646 | 2.1048 | 2.2357 | 1.5954 |
| SAMMed2D 5 points | 0.2575 | | | | | | | | 79.5836 | | | | | | | |
| SAMMed2D 16 points | 0.4484 | | | | | | | | 27.4014 | | | | | | | |
| SAMMed2D 32 points | 0.3478 | | | | | | | | 38.7130 | | | | | | | |

**Table S19. Comparison results of proposed method based on automatic prompt and SAM Med2d based on manual points prompt in REFUGE dataset.**

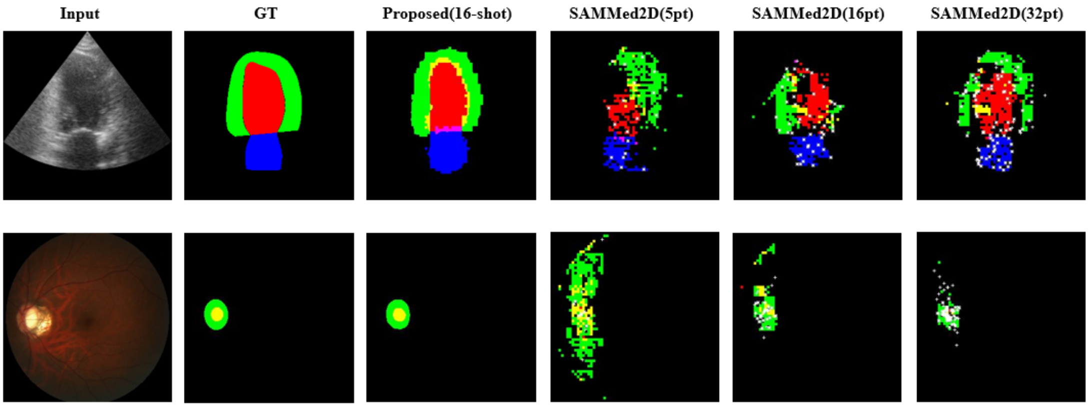


**Figure S6. | Comparison results of proposed method based on automatic prompt and SAM Med2d based on manual points prompt.**
